# Does intraspecific competition promote variation? A test via synthesis

**DOI:** 10.1002/ece3.1991

**Published:** 2016-02-12

**Authors:** Andrew W. Jones, David M. Post

**Affiliations:** ^1^ Department of Ecology and Evolutionary BiologyYale University165 Prospect StreetNew HavenConnecticut06511USA; ^2^ Department of BiologyWoods Hole Oceanographic Institution266 Water StreetWoods HoleMassachusetts02543USA

**Keywords:** adaptive divergence, interaction strength, intraspecific competition, niche width, resource depletion

## Abstract

Competitive diversification, that is, when increasing intraspecific competition promotes population niche expansion, is commonly invoked in evolutionary studies and currently plays a central role in how we conceptualize the process of adaptive diversification. Despite the frequency with which this idea is cited, the empirical evidence for the process is somewhat limited, and the findings of these studies have yet to be weighed objectively through synthesis. Here, we sought to fill this gap by reviewing the existing literature and collecting the data necessary to assess the evidence for competition as a diversifying force. Additionally, we sought to test a more recent hypothesis, which suggests that competition can act to both promote and inhibit dietary diversification depending on the degree to which a consumer depletes its resources. The surprising result of this synthesis was that increasing competition did not have a mean positive effect on population‐level diet breadth or the degree of individual specialization. Instead, we found that increasing intraspecific competition had a restricting effect on population‐level diet breadth in as many cases as it had a diversifying effect. This wide disparity in the effect of competition on consumer diet variation was negatively related to a metric for consumer resource depletion. Altogether, these findings call into question a long‐standing assumption of basic evolutionary models and lend some support to recent theoretical predictions. Specifically, these findings support the idea that competition is primarily diversifying for species with a small effect (per unit biomass) on their resources and that resource depletion limits the diversifying effect of competition for consumers with larger ecological effects.

## Introduction

A common assumption shared by current models of adaptive divergence and adaptive radiation is that increasing intraspecific competition should promote population‐level niche expansion and variation among its constituent individuals (Schluter 2000; Yoder et al. 2010; Nosil 2012). According to this paradigm, increasing consumer density is expected to reduce the availability of resources, causing individuals to utilize a greater variety of resources and potentially leading to divergent selection on traits associated with resource utilization. This general phenomenon has been described as a population traveling “down the slopes of its adaptive peak” (Svärdson 1949) and has been invoked in a wide variety of adaptive scenarios for an extended period (Mayr 1926; Pfennig and Pfennig 2012). Considerable theoretical support for this process exists (Roughgarden 1972; Futuyma and Moreno 1988), however, empirical support is more limited (but see Bolnick 2001). A growing number of empirical tests do not find support for competitive diversification, and indeed, some suggest competition may inhibit diversification (Schindler et al. 1997; Haley et al. 2011; Svanbäck et al. 2011; Jones and Post 2013; Parent et al. 2014). It is likely that this phenomenon is more complex than previously anticipated and that further work is needed to extend what has been a simplistic but useful conceptual construct.

To begin the process of clarifying the relationship between intraspecific competition and intraspecific dietary variation, a commonly assayed form of intraspecific variation, we collected and assessed the existing evidence via a focused meta‐analysis. We use a taxonomically focused sample of the existing empirical results to test two broad predictions about the effects of intraspecific competition on a consumer population's dietary niche width: The first prediction is that variation within a population should increase with increasing intraspecific competition (hereafter the competitive diversification [CD] hypothesis) and the second prediction is that the effect of competition on niche width is moderated by the degree to which a consumer depletes its resources (Svanbäck and Bolnick 2005; Abrams et al. 2008; Jones and Post 2013). Abrams et al. (2008) articulated a nonlinear relationship between competition and dietary diversification, where competition could be both diversifying in some instances and restricting in others. As predicted by the CD hypothesis, Abrams et al. argued that at low levels of competition, a consumer population uses only preferred prey, while at moderate levels of competition, the reduced abundance of preferred resources drives individual consumers to use both preferred and nonpreferred resources leading to population niche expansion. However, these authors also suggest an alternate scenario for when populations experience intense levels of intraspecific competition. Specifically, they suggest that at these higher levels of competition, when preferred resources are more thoroughly depleted, only the remaining nonpreferred resources are consumed, effectively restricting population‐level dietary niche width. Building on Abrams et al. (2008), Jones and Post (2013) suggested that this hump‐shaped relationship results from competition being a function of both the consumer density and an estimate of their per capita (or per‐unit‐biomass) effect on their resources. This verbal model suggests that individual consumer species may experience only a portion of a roughly unimodal relationship between competition and dietary variation and that the region they experience will be determined by their propensity to deplete resources. Hereafter, we refer to this alternate hypothesis as the intermediate competitive diversification (ICD) hypothesis.

An important consideration in testing these hypotheses is that variation in the diets of a population's constituent individuals can manifest and can be measured in a number of ways. Individuals can have diets that are more generalized (and mirror population‐level diet variation) or are restricted to a subset of the population diet (see Bolnick et al. 2003). This variation is especially important to consider in this context, as a restriction in population niche width could potentially coincide with increasing variation among individuals. While this is unlikely, given that most populations with greater niche width exhibit increased individual specialization (Van Valen 1965; Bolnick et al. 2007), we sought to investigate this possibility.

In order to construct a dataset with which to assess these ideas, we strategically restricted our data collection efforts to studies that focused on species of fish. We chose this group *a priori* because it contains many species in which adaptive divergence is thought to have occurred (Robinson and Wilson 1994), and because intraspecific competition is often hypothesized as a likely driver of divergence in this group (Schluter 2000; Bolnick 2004). Intraspecific competition also has a range of effects, both at the population and individual levels, on dietary variation in fish (Schindler et al. 1997; Svanbäck and Persson 2004; Svanbäck and Bolnick 2007). Fishes as a group are also known to exhibit varied top‐down effects on prey abundance, ranging from those that have large effects on their resources (Brooks and Dodson 1965; Palkovacs and Post 2009), to others that have barely detectable direct effects on their resources. Focusing on fishes also minimized issues relating to standardizing consumer effects across ecologically and physiologically disparate taxa and provided a sufficient sample of studies for analysis, which was not possible for other taxonomic groups.

To assess the effect of increasing density (and competition) on a population's niche width, we employed a standard response ratio measure of effect size (Hedges et al. 1999; Koricheva et al. 2013), and to estimate an overall summary mean effect size, we used a standard mixed model analysis (Viechtbauer 2010). The prevailing CD hypothesis predicts a positive summary mean effect of density (and competition) on population niche width. Testing the specific predictions of the ICD hypothesis required us to calculate both a measure of per‐unit‐biomass prey depletion and a measure of how consumer density affects diet variation. Our measure of per‐unit‐biomass effect of a consumer, hereafter referred to as resource depletion (RD), is functionally and conceptually similar to Paine's classic empirical formulations of interaction strength (Paine 1992; Power et al. 1996; Laska and Wootton 1998; Wootton and Emmerson 2005). Our RD index estimates the net reduction in resources by a consumer and has the advantage of encompassing differences in resource growth rate (Abrams 2001; Novak and Wootton 2010). Thus, it was appropriate for quantifying a per capita or per‐unit‐biomass ability of a predator to deplete its prey, which is essential to testing the existing alternate ICD hypothesis. We also constructed a metric for how a standardized change in consumer density affects a consumer's dietary niche width. This metric essentially describes whether increasing consumer density (and increasing intraspecific competition) has a diversifying or restricting effect on the diet of the consumer of interest.

After calculating these metrics for each dataset, we tested three specific predictions related to the ICD hypothesis using mixed model analyses: (1) We expected that the summary mean effect size should be near zero or at least not strongly positive (as predicted by the CD hypothesis); (2) increasing consumer density would have a positive effect on population‐level dietary variation in consumers with lower RD values and a negative effect on dietary variation in consumers exhibiting high RD values; (3) when comparisons were made across consumer taxa, the general relationship between intraspecific competition and niche width is roughly unimodal. Finally, we expected that individual‐level dietary variation would roughly mirror population‐level dietary variation (Bolnick et al. 2007).

## Methods

### Data collection

We performed a focused literature search to collect a broad sample of consumer resource studies with which to test our hypotheses. We accomplished this by searching collections of papers citing two foundational studies of direct top‐down effects, Paine (1980) and Sih et al. (1985). We chose this area of research because it is rich in both observational and manipulative empirical studies that explore the top‐down effects of consumers, and studies of this nature often involve the manipulation of a focal consumer's density. In addition, we opportunistically added studies from the most recent literature that explores the process of competitive diversification.

Primary lists of citation to search were obtained from Web of Science on 15 November 2015. Opportunistic literature collection occurred through the date of submission. From these searches, we compiled papers that met the following five criteria. First, studies needed to be empirical and related to consumption. Second, the studies needed to manipulate or report a difference in density of a single consumer population. Studies of multiple consumer species were excluded to avoid the potentially confounding effects of multiple consumers on resource populations. Third, studies needed to report consumer biomass density such that it could be standardized (to g/m^2^) for novel metric calculation. Fourth, studies needed to report diet information such that the diet breadth of the consumer could be compared between density treatments. Finally, the effect of the consumer on one or more primary resources needed to be reported so that the RD of the consumer could be estimated. Data were compiled from all studies that met our criteria for inclusion. When necessary, information was retrieved from digitized graphs.

The datasets used this article have been uploaded as part of the Supporting Information. In total, over 1500 studies were accessed and assessed for the appropriate data. Studies fell into two categories: (1) observational studies where consumer density varied spatially or temporally and (2) experimental studies where consumer density was altered at one or more levels. When multiple data points were available, multiple data points were collected. For experimental studies, low and high treatment designations followed the assignment of the authors. For observational studies, specific data points were designated as either low‐ and high‐density treatments and included in the calculation of means as such.

Searching a wide variety of studies cast a wide net and ensured that our studies came from taxonomically diverse set of consumers. However, this also meant that the vast majority of studies we surveyed did not meet the criteria for inclusion in our analysis. The primary reason studies were excluded from the analyses varied subtly between the two literatures (Fig. S1). In both cases, though, studies were excluded primarily because they either were not an empirical study (e.g., a review or opinion) or because they did not include sufficiently diet information for the calculation of niche width.

### Effect size calculation

All statistical analyses for this study were conducted in R (R Development Core Team 2014). To compare the effect of increasing competition on population niche width, we used the R package metafor (Viechtbauer 2010) to estimate an effect size for each included study. For this, we used a mean difference approach, and utilizing mean population niche width for consumers at high and low consumer densities, we calculated a log response ratio (Hedges et al. 1999). Other metrics of effects, such as standard mean difference (Bonett 2009), produced qualitatively indistinguishable results. Next, we calculated a summary mean effect size, using the log ratio effects sizes and associated variability derived from each set of comparisons via a mixed effects meta‐analysis (Viechtbauer 2010; Koricheva et al. 2013). Similar metrics were calculated for estimates of individual specialization to assess the effect of competition on variation among individuals within a population.

### Calculation and analysis of novel comparative metrics:

In addition to estimating the summary mean effect of competition on population niche width, we were interested in utilizing more mechanistic metrics to test recent theoretical predictions. Historically, population‐level resource depletion (often referred to as interaction strength) has been calculated in a number of ways. Here, we developed a metric similar to Paine's index (1992), which has been used in many experimental studies to estimate per capita interaction strength (Fagan and Hurd 1994; Berlow et al. 1999). Paine's index is typically calculated as:

(1) $$\mathrm{PI}\;=\;\frac{\left(N_{+P}-N_{-P}\right)}{PN_{-P}}$$

where N_+P_ is the resource abundance in the presence of a consumer, N_−P_ is the resource abundance in the absence of a consumer, and P is the abundance of the consumer (Novak and Wootton 2010). Paine's index has typically been used to estimate resource depletion in situations in which a consumer is present or absent. Our metric of resource depletion (RD) is similar to Paine's index but modified slightly to compare effects across situations in which the focal consumer varies in density:

(2) $$\mathrm{RD}\;\;=\;-1*\;\frac{\left(N_{h}-N_{l}\right)}{\left(P_{h}-P_{l}\right)N_{l}}$$

where *P*_*h*_ and *P*_*l*_ are the mean high and low (respectively) consumer biomass densities in units of (g/m^2^), and *N*_*h*_ and *N*_*l*_ are the mean resource values at high and low consumer densities, respectively. There are three primary differences between these measures: (1) the *P* in the denominator of the original metric (used to standardize effects to a per capita value) becomes *P*_*h*_–*P*_*l*_ (to standardize the effects to per‐unit‐biomass [g/m^2^]), (2) we multiply the metric by −1 in an effort to make it more intuitive, and 3) we utilized study means of high and low density rather than paired high and low density replicates.

Thus, higher values of RD indicate a consumer population with a larger per biomass effect on its resources, while lower values indicate a consumer with a smaller per biomass effect on its resources. For each comparison, RD was calculated for each resource group reported from the study. Although mean RD and variation in consumer RD across resource groups is of interest, we focused only on the prey group that exhibited the greatest change in abundance. We chose to focus on these groups because most studies report the effects of consumers on only a limited set of resources (potentially biasing estimates of mean RD).

Population‐level diet variation in the form of Levins' B (Levins 1968) was calculated from the reported population‐level diet proportions as:

(3) $$B\;=\;\frac{1}{\sum p_{i}^{2}}$$

where *B* is the measure of population diet breadth, and *p*_*i*_ is the fraction of total diet mass represented by resource *i*. Higher values of *B* indicate populations with more varied diets. Because the number of diet categories included in a given study influences Levins' B metric, we normalized values within studies to values between zero and one using feature scaling. For each study, we then calculated the change in standardized niche width relative to the mean change in density as:

(4) $$\frac{\Delta N}{\Delta D}\;=\;\frac{B_{h}-B_{l}}{P_{h}-P_{l}}$$

where *B*_*h*_ and *B*_*l*_ are the mean diet breadth of the consumer population at high and low consumer densities, and *P*_*h*_ and *P*_*l*_ are the mean high and low consumer biomass densities in units of (g/m^2^). ∆D represents the change in density, and ∆N represents the change in niche width. Thus, positive values of the metric ∆N⁄∆D indicate a diversifying effect of competition, and negative values of ∆N⁄∆D indicate a restricting effect of competition. We then assessed the importance of RD to ∆N⁄∆D (the relationship between mean niche width and mean density) by comparing the fit of mixed models. For each model, the fixed effects included the maximum RD, log_10_‐consumer biomass, as well as the type of study (whether the study was observational or manipulative) and a random intercept for study identity. We assessed the importance of RD to ∆N⁄∆D by fitting a model that included RD to one that did not include this term.

In order to estimate the strength of intraspecific competition, we first normalized (again using feature scaling) both the consumer density and maximum RD derived from each comparison, and then multiplied these two values together. While this is a crude estimate of intraspecific competition, it captures the essence of recent theoretical work that highlights the idea that intraspecific competition is a function of a consumer population's raw biomass density and per‐unit‐biomass effect size. We then used this metric to fit a series of mixed models, utilizing our standardized metric of niche width (standardized Levins' *B*) as a response variable. In these models, we included our measure of intraspecific competition as a fixed effect and a random intercept for each study. Model comparisons were then used to assess the general relationship between intraspecific competition and niche width. Specifically, we compared one model where IC was included as a linear term, one model where it was included as a quadratic term, and a final null model where it was omitted.

We focused our inclusion criteria and analyses on population‐level niche width because it is the simplest and most commonly reported form of intraspecific variation. However, intraspecific variation could also manifest as variation among individuals (individual specialization – *sensu* Bolnick et al. 2003), and thus, when these data were available, they were also collected. The metrics reported in studies varied, but each represented increasing specialization as a decreasing number. Because of differences in how these metrics were reported, we did not include individual specialization in our calculations of comparative metrics, but instead estimated log response ratios in a manner identical to that used for population niche width.

Mixed models of the comparative metrics were fit using maximum likelihood in the lme4 R package (Bates et al. 2014). In all analyses, we utilized multiple studies of a single species as independent data points because of the limited number of studies available. Additionally, many of the studies utilizing similar species are from distinct locations and represent the efforts of distinct sets of investigators. Significance was assessed using the accompanying lmerTest R package (Kuznetsova et al. 2014), which utilizes Satterthwaite's approximation to estimate degrees of freedom. Model fit was compared using AIC (Sugiura 1978), and the likelihood ratio test was implemented in the lmerTest R package (Kuznetsova et al. 2014).

## Results

### Overall effect size

**Figure 1:**
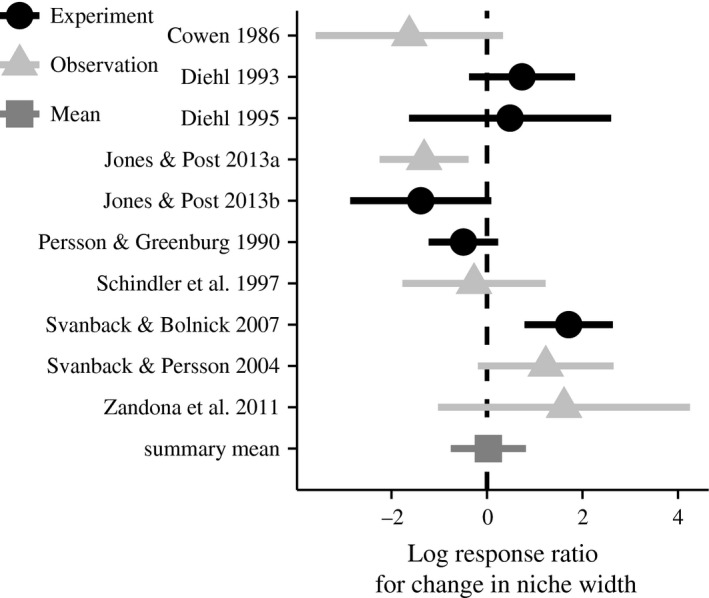
Effect sizes suggest no mean effect of competition on population‐level niche width. The log response ratio effect sizes for the studies included in the analysis. Mean effect sizes are coded by the type of study, with dark circles representing experimental studies and lighter triangles representing observational studies. Error bars represent 95% confidence intervals. The summary mean effect size derived from the metafor‐based mixed model analysis is shown at the bottom.

The prevailing CD hypothesis predicts a positive mean effect size; however, here we found that the summary mean effect size of increasing density (and competition) on population niche width was −0.12 (SE = 0.92) and, therefore, did not differ from zero. There was, however, significant variation among studies (*Q* = 28.67; *P* < 0.001; Fig. 1), with mean effects in half of the studies (5/10) indicating increasing population niche width in response to increasing competition, and half indicating decreasing niche width in response to increasing competition. Both of these findings fit well with the general predictions of the ICD hypothesis, which suggest a mean summary effect near zero, resulting from the averaging of studies with substantial positive and negative effects. There were no significant differences in effect size between experimental and observational studies. Estimated effect sizes for estimates of individual specialization also varied among studies, with a summary mean effect size near zero (Fig. S2A). As we expected, estimated effect sizes for individual specialization were tightly correlated with effect sizes for population‐level variation (*R* ^2^ = 0.75, *P* < 0. 05, Fig. S2B).

### Specific prediction of the ICD hypothesis

**Figure 2:**
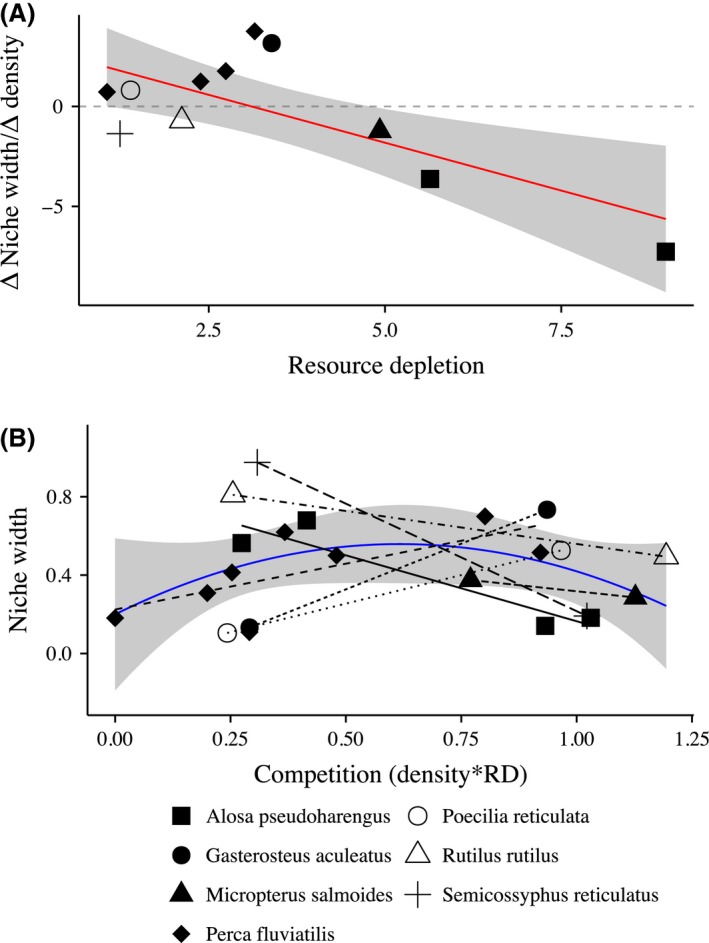
Metrics suggest resource depletion influences population niche width. (A) The relationship between a metric that describes how a consumer population's niche width changes with density, and the degree to which consumers deplete their resources. Values above dashed zero line represent studies where increasing competition among conspecific consumers had a diversifying effect on their population‐level dietary variation. Values below zero represent cases where increasing competition had a restricting effect on dietary variation. Each point represents a single study. Shapes represent different species. The fitted line (red) represents the reduced model including only RD. (B) The more general relationship between niche width and a metric for competition. Both niche width and densities have been normalized to values between zero and one. Shapes and line types represent each species from which the data were derived. The quadratic fitted line (blue) suggests a unimodal relationship and supports predictions of the ICD hypothesis. In both figures, the gray ribbon represents a 95% confidence interval for the simplified fitted lines (red and blue).

Consistent with the specific mechanistic prediction of the ICD hypothesis, we found that consumer resource depletion (RD) had a significant negative effect on the relationship between dietary population‐level dietary variation and density (Fig. 2A, Table 1). When consumers had a small effect on their resources, we observed positive (diversifying) values of ∆N⁄∆D, and when consumers had a large effect on their resources, we observed negative (restricting) values of ∆N⁄∆D. Other factors included in the mixed model analysis, such as consumer biomass, accounted for little of the variation in our model (Table 1). Mixed models fit to assess the shape of the relationship between niche width and our metric of intraspecific competition (Fig. 2B) suggested that the quadratic model provided a slightly better fit to the data (χ^2^ = 2.73, *P* = 0.09) than the linear model. While this difference was not significant at the *P* < 0.05 level, it is highly suggestive, especially given the limited number of studies included in the analysis. This finding suggests that the maximum for the quadratic function was at a positive intermediate value of our metric for intraspecific competition and that the general relationship between niche width and competition is likely to be roughly unimodal.

**Table 1:** The effect of resource depletion on our metric for change in population dietary niche width with increasing density

Fixed effects	df	*t*‐value	*P*‐value
Intercept	10.15	2.01	0.07
Resource depletion	10.32	−2.99	**0.01**
log_10_ (biomass)	10.05	−0.54	0.60
Study type	9.95	0.05	0.96

Random effects	Variance	SD
Intercept by study ID	4.05	2.01
Residual variance	0.39	0.63

Here, we show the result from the full mixed model. Significant results are shown in bold. A likelihood ratio based on the comparison of the full model to a null model missing fixed effect for resource depletion suggested that resource depletion had a significant effect (χ^2^ = 5.69, *P* = 0.02) on the metric constructed to describe the relationship between diet variation and increasing density (∆N⁄∆D).

## Discussion

The idea that competition among conspecifics acts to promote variation within a population is truly engrained in the thinking of ecologists and evolutionary biologists (e.g., Yoder et al. 2010), yet it is supported with a limited set of empirical examples (e.g., Bolnick 2001; Svanbäck and Bolnick 2007). Given the pervasiveness of this idea in the literature, we were surprised that so few studies contained the data necessary to assess the effect of increasing competition on dietary variation. In the studies that included necessary data, increasing competition had a restricting effect (decreasing population niche width) in as many cases as it has a diversifying effect. These findings provide limited support for the classic CD hypothesis and call into question the idea that competition is principally a diversifying force.

The results of this study are instead most congruent with the predictions of the more recent ICD hypothesis. Specifically, results from our first analysis suggest that while the mean effect did not differ from zero, there was significant heterogeneity in effect sizes among the included studies, and much of that heterogeneity is explained by the effect of a consumer on its prey (RD). The negative relationship between change in niche width with density (∆N⁄∆D) and resource depletion (Fig. 2A) also meshes well with the mechanistic predictions of the ICD hypothesis. Together these lines of evidence seem to support the general predictions of Abrams et al. (2008) and Jones and Post (2013), which intraspecific competition acts to both enhance and restrict diversity.

Our second set of model comparisons also suggested results in line with the ICD hypothesis. While this work is derived from a limited number of studies, it produced a highly suggestive unimodal nonlinear relationship between the metric of intraspecific competition (which incorporated RD) and population niche width (Fig. 2B). Moreover, we found similar trends in individual specialization, with a strong correlation between changes in individual dietary variation with density and changes in population‐level dietary variation with density. This finding matched our expectations as well as patterns that have previously been reported (Bolnick et al. 2007).

The current evolutionary paradigm assumes that increased intraspecific competition within consumer populations — which could result from density compensation — is a diversifying force that typically causes niche expansion and could lead to disruptive selection and adaptive divergence (Schluter 2000; Yoder et al. 2010; Nosil 2012). Our results indicate that increased competition (via increasing density) among conspecific consumers may have this effect under specific circumstances (Bolnick 2001; Svanbäck and Persson 2004; Tinker et al. 2008), but that in many other cases, it can instead have a restricting effect on population‐level dietary variation. One such situation, where competition leads to dietary restriction rather than diversification, occurs when consumer taxa exhibit large ecological effects on their resources. We see clear evidence of this occurring in a number of studies in the dataset. Consumers well‐known to have larger effects on their resources [i.e., alewife (Brooks and Dodson 1965; Wells 1970; Post et al. 2008), largemouth bass (Power et al. 1985; Schindler et al. 1997), and roach (Persson and Greenberg 1990)] exhibit the most negative values of ∆N⁄∆D. We also see suggestions of a negative relationship between consumer RD and ∆N⁄∆D in studies of fishes for which there were not sufficient data to be included in our analysis (e.g., Hambright and Hall 1992), and in a broader sample of taxonomic groups for which the number of studies was too few to perform a formal analysis (Lomolino 1984; Thurber and Peterson 1993).

Given the limited number of studies identified by our extensive literature search, it is clear that determining the exact shape of the relationship between competition and dietary variation will require additional empirical studies. Such studies should be viewed as a research priority because the exact shape of this relationship, especially where the inflection point lies, has important implications for our understanding of the process of adaptive divergence in nature. If the inflection point occurs at high levels of competition (i.e., levels that most species do not commonly experience), then it suggests that a diversify effect of competition may be common. Conversely, if the inflection point occurs at low levels (i.e., those more commonly experienced), then it would suggest that competition is more commonly a restricting force.

In addition to determining the more general shape between competition and population‐level variation, more work is needed to constrain the shape of the relationships for each species. One intriguing possibility, which is implied by recent theory (Svanbäck and Bolnick 2005), is that the relationship may be hump shaped for each species. With our results, we were unable to determine whether the shape of this more general relationship is a result of a combination of linear relationships for each species or that each species exhibits a nonlinear relationship and the linear pattern we observe for each species is a result of observations over a limited subset of the full set of possible densities. While more work remains, the fact that the relationship is clearly not a flat or positive increasing function—as would be expected by the CD hypothesis—has a number of important implications for understanding the forces shaping intraspecific variation in consumers and expectations for drivers of adaptive diversification.

Based on these findings, the process of sympatric, ecologically‐driven diversification, as it is currently envisioned (Schluter 2000; Yoder et al. 2010; Nosil 2012), is unlikely to occur in consumers that have strong depleting effects on their resources (high values of RD). Conversely, while strong ecological effects may limit sympatric diversification, they may enhance the probability of divergence between allopatric populations. For example, if the identity of the resources that high RD consumers restrict themselves to varies among populations (e.g., one population is restricted to a large‐bodied resource, while the other is restricted to a small‐bodied resource), divergence could be promoted between separated populations (Thompson 2005; Benkman 2013). Ultimately, the RD value of a consumer may determine how intraspecific variation is balanced within and among populations. This balance is likely to affect patterns of diversification and to have important implications for the conservation and management of populations. This is because variation within and among populations is thought to be important for population stability (Schindler et al. 2010; Bolnick et al. 2011), and presence or absence of variation within a population can have a number of important ecological effects (Palkovacs and Post 2008).

While we have focused this analysis on the top‐down effects of consumers, other factors such as consumer mortality (Abrams et al. 2008) and bottom‐up forces (e.g., Edwards et al. 2010) could shape the relationship between competition and population‐level dietary variation. For example, ecosystem productivity and resource diversity may play a role in shaping consumer RD (Hillebrand and Cardinale 2004) and thereby could affect the relationship between intraspecific competition and dietary variation. Indirect effects can shape the identity or availability of resources and have similar effects on the process of competitive diversification (Walsh and Reznick 2008). Additionally, recent studies provide some support for the idea that increased dietary variation promotes consumer fitness (Lefcheck et al. 2012), suggesting that forces promoting population‐level diet variation could feedback to affect consumer population dynamics or selection. This type of feedback could lead to evolutionary changes (an eco‐evolutionary feedback *sensu* Post and Palkovacs 2009; Schoener 2011; Estes et al. 2013), altering consumer RD and potentially enhancing community stability (Kondoh 2003). Finally, it is worth noting that population‐level consumer RD is nonlinearly associated with the strength of selection experienced by resource taxa (Benkman 2013), and selection on resource populations could feedback to shape consumer dietary variation in a number of ways.

In dealing with aspects of the niche, it is also important to consider that the niche is typically described as having two aspects: an expansive fundamental niche comprised of all the resources suitable for existence and the realized niche which describes the subset of resource that are actually utilized (Hutchinson 1957). Here, we are dealing exclusively with the realized niche. It is possible that the fundamental niche of a population responds differently to intraspecific competition. For these reasons, we suggest that further work explicitly considering the fundamental niche would be valuable.

Here, we aimed to enhance the connections between the topics of adaptive divergence and the top‐down effects of consumers, which have not been strongly linked historically (but see Wilson and Turelli 1986; Svanbäck and Bolnick 2005). The literature on adaptive divergence has substantial value in that it explores the processes responsible for the generation of variation within populations (Schluter 2000). The literature on the effects of consumers explores the ecological impacts of consumer populations on the abundance of organisms at lower trophic levels, and how these interactions shape community and ecosystem properties (Paine 1980). By further integrating these two subjects, we aimed to improve our understanding of the forces producing and maintaining biological variation at multiple levels of organization. That we were able to find so few studies that met the minimal criteria for inclusion suggests that the gap between these two fields is substantial. Future studies should attempt to fill this void by more explicitly considering the links between competition, diet variation, and the top‐down effects of a consumer.

## Conflict of Interest

None declared.

## Data Accessibility

The data that were collected and utilized for this study can be viewed via DRYAD entry doi: http://dx.doi.org/10.5061/dryad.h61dj

## Supporting information


**Figure S1.** Primary reasons for excluding studies from our analyses.

**Figure S2.** Response of individual specialization to increasing competition.

**Data S1.** File containing the data collected as well as data summaries.

**Data S2.** R Code used for our analyses and figure creation.
