# Supplementary figures and images for: Does intraspecific competition promote variation? A test via synthesis
Source: Ecol Evol. 2016 Feb 12;6(6):1646–55. doi: 10.1002/ece3.1991 (PMC4801976; doi:10.1002/ece3.1991)

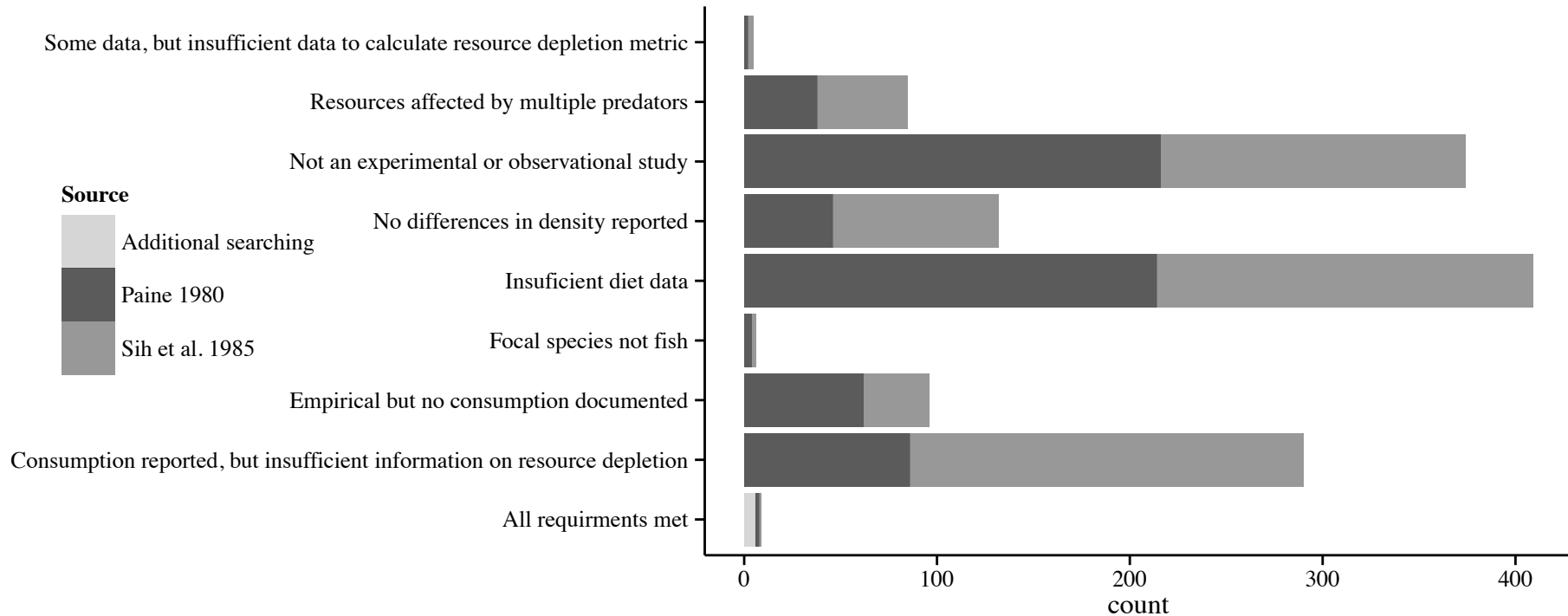

Supplement: Supplementary file 1 — Figure S1. Primary reasons for excluding studies from our analyses. [file ECE3-6-1646-s001.pdf]

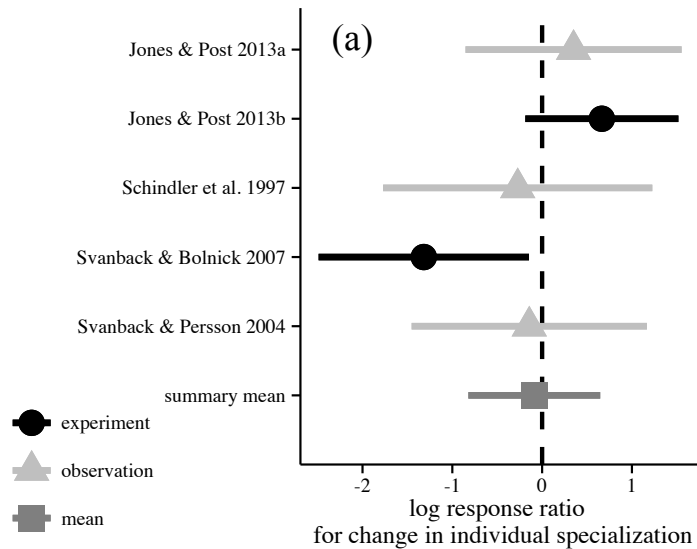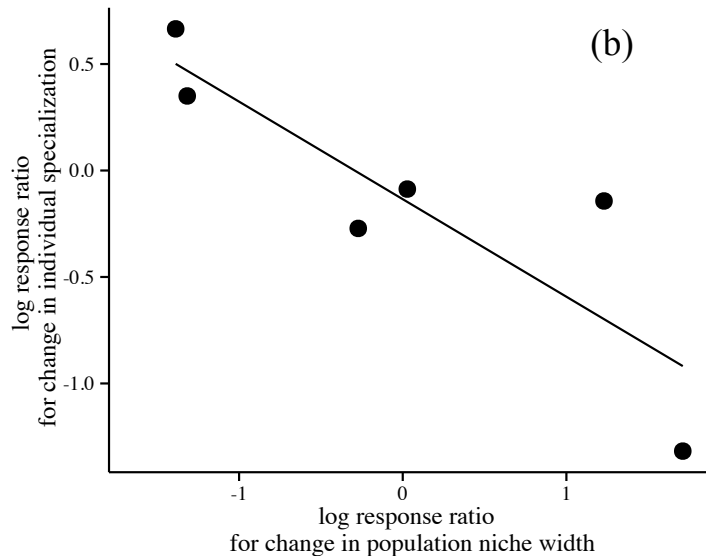

Supplement: Supplementary file 2 — Figure S2. Response of individual specialization to increasing competition. [file ECE3-6-1646-s002.pdf]
