# Supplementary material for: Does intraspecific competition promote variation? A test via synthesis
Source: Ecol Evol. 2016 Feb 12;6(6):1646–55. doi: 10.1002/ece3.1991 (PMC4801976; doi:10.1002/ece3.1991)
Supplement: Supplementary file 5 [file ECE3-6-1646-s005.docx]

**Supporting Information**

**Does intraspecific competition promote variation? A test via synthesis.**

Andrew W. Jones, David M. Post

**Figure S1: Primary reasons for excluding studies from our analyses.** The primary reason studies were excluded from our analyses. Often studies could be excluded for multiple reasons, however only a single reason for exclusion was recorded.

**Figure S2: Response of individual specialization to increasing competition.** (a) The log response ratio effect sizes estimated for each study included in the analysis. Together these data suggest no mean effect of competition on individual specialization. Mean effect sizes are coded by the type of study, with dark circles representing experimental studies and lighter triangles representing observational studies. Error bars represent the 95% confidence intervals. The summary mean effect size derived from the metafor based mixed model analysis is shown at the bottom. Note that decreasing values of correspond to increasing levels of individual specialization. (b) Effect sizes for population-level niche width and individual specialization are correlated. The correlation shown suggests that when population niche width was larger there was a higher degree of variation among individuals. Again decreasing values correspond to increasing levels of individual specialization.

**File containing the data collected as well as data summaries.** A file containing the raw data collected for the meta-analysis as well as data summaries. A data file titled “Jones & Post Data.xlsx” with sheets named to indicate which portion of the analysis they were used in. These include: 1) A sheet titled “Jones Post Studies Surveyed.csv” containing a list of more than 1500 studies surveyed. The reasons for excluding each study are listed in the second column. Often studies were excluded for multiple reasons. In this case, only one reason for exclusion is listed. 2) A sheet titled “Data Collected.csv” containing the data derived from the included studies. Often, data was digitized from graphs. 3) A sheet titled “Data metaphor PNW.csv” containing summary statistics derived from the included studies for the population-level niche width calculations. Columns include the mean and standard deviation values of the standardized Levins’ B metric for low and high-density treatments. 4) A sheet titled “Data metaphor IS.csv” containing summary statistics derived from the included studies for the individual specialization calculations. Columns include the mean and standard deviation values of the standardized individual specialization metrics for low and high-density treatments. 5) A sheet titled “Data figures.csv” containing summary statistics derived from the included studies. Metrics calculated from the comparison of low and high-density treatments are reported for high-density treatment only. Each sheet must be saved as an independent .csv file of the same name to be used with the R code.

**R Code used for our analyses and figure creation.** The R code needed to carry out the analyses we report. We include the code needed to create the figures as well. See annotations throughout the script for a very brief description of each step.
